# Zn‐Assisted Mg Ion Transport in Spinel Oxide Cathodes: Insights From Neural Network Simulations

**DOI:** 10.1002/asia.202500908

**Published:** 2025-12-15

**Authors:** Riku Nakahara, Naoto Tanibata, Hayami Takeda, Masanobu Nakayama, Kohei Shimokawa, Tetsu Ichitsubo

**Affiliations:** ^1^ Department of Materials Science and Engineering Nagoya Institute of Technology Nagoya Japan; ^2^ Frontier Research Institute for Interdisciplinary Sciences Tohoku University Sendai Japan; ^3^ Institute for Materials Research Tohoku University Sendai Japan

**Keywords:** Mg ion batteries, molecular dynamics, neural network potential

## Abstract

We investigated Mg insertion and ion transport in defect spinel Mg*_x_* ZnMnO_3_ (0 ≤ *x* ≤ 1) using a neural network potential combined with molecular dynamics and genetic algorithm–based structure exploration. The diffusion coefficients showed a strong composition dependence: both stoichiometric spinel (*x* = 0.25) and rock‐salt (*x* = 1.0) exhibited extremely low conductivity (<10^−10^ cm^2^ s^−1^), whereas enhanced diffusivity appeared in the cation‐deficient (*x* < 0.25) and cation‐excess (0.25 < *x* < 1.0) regions, reaching 3.97 × 10^−7^ cm^2^ s^−1^ at *x* = 0.50. Zn^2+^ consistently diffused faster than Mg^2+^, and both species migrated along the same 8a–16c–8a channels, suggesting possible concerted hopping interactions. Thermodynamic analyses revealed that the vacancy‐driven spinel region (*x* < 0.25) is stabilized as a solid solution, whereas in 0.25 < *x* < 0.84 a biphasic spinel–rock‐salt coexistence is favored. The precipitation of stoichiometric phases (*x* = 0.25, 1.0) may hinder Mg transport and reduce capacity, but the low energy‐above‐hull values suggest solid‐solution pathways are kinetically accessible. These findings indicate that Zn plays a dual role by stabilizing tetrahedral sites and enhancing Mg mobility, providing design guidelines to achieve both high voltage and improved diffusivity for magnesium battery cathodes.

## 1 Introduction

With the increasing adoption of renewable energy, the demand for large‐scale energy storage systems to mitigate the instability of power supply and demand is rapidly expanding. Lithium‐ion batteries (LIBs), which play a central role in this field, have demonstrated outstanding performance in portable electronics and electric vehicles (EVs) [1]. However, they face challenges such as the use of scarce elements like lithium and cobalt, as well as high costs. Furthermore, although the use of metallic lithium as the anode holds promise for achieving a large increase in capacity, safety concerns associated with short‐circuiting due to dendrite formation present obstacles to large‐scale implementation. To overcome these issues, intensive research has been directed toward next‐generation rechargeable batteries that utilize multivalent metal‐ion carriers, which are abundant in resources and offer superior safety.

Among them, rechargeable magnesium batteries (RMBs) are particularly attractive, as magnesium metal anodes exhibit a low reduction potential of −2.37 V versus SHE [2], while simultaneously suppressing dendrite formation even when metallic Mg is used as the anode [3, 4, 5]. In addition, magnesium is abundant in the Earth's crust and possesses a high volumetric capacity of 3833 mA h cm^−3^, making it an extremely promising candidate for future large‐scale energy storage applications. For cathode materials, intercalation‐type oxides are preferred due to their potential for high operating voltages [6]. However, the strong electrostatic interactions between divalent Mg^2+^ ions and oxide anions impose a large migration energy barrier for Mg^2^⁺ ion hopping, which severely limits Mg^2^⁺ diffusivity within the cathode [7, 8].

Oxide spinel‐type cathode materials have attracted considerable attention as promising candidates for RMB cathodes, since their three‐dimensional ionic diffusion pathways, constructed by tetrahedral cation sites and octahedral vacant sites (8a, 16c, and 16d crystallographic sites, assuming *Fd3m* symmetry), are considered to be relatively favorable for Mg diffusion [9, 10, 11]. For example, we confirmed reversible Mg insertion/extraction reactions in spinel‐type MgM_2_O_4_ (M = Mn, Fe, Co) materials, where Mg^2^⁺ can be topochemically inserted to form the rock‐salt phase Mg_2_M_2_O_4_. More recently, we demonstrated that nanosizing of these materials enables charge–discharge operation even at room temperature. On the other hand, the rock‐salt phase Mg_2_M_2_O_4_ exhibits a highly stable structure in which all cations occupy octahedral sites, thereby suppressing reversibility and causing deterioration of cycling performance [12]. To address this issue, we investigated spinel‐type ZnM_2_O_4_ materials, in which Zn preferentially occupies tetrahedral sites instead of Mg, and found indications of improved cycling performance [12]. Furthermore, by designing a defect spinel structure of ZnMnO_3_ incorporating cation vacancies at the 16d sites and partially at the 8a sites, we demonstrated that the material delivers both high cycle stability over more than 100 cycles and a combination of high operating voltage (≈2.5 V vs. Mg^2^⁺/Mg) and high capacity (≈100 mA h g^−1^) [6]. This defect spinel structure not only extends the compositional stability range of the spinel phase through the tetrahedral site preference of Zn and the introduction of vacancies [12], but also enhances the theoretical capacity through the increase of Mn oxidation states [6].

In the aforementioned studies [6, 12], first‐principles calculations combined with structural optimization using a genetic algorithm revealed that, in the Mg*_x_* ZnMnO_3_ spinel material capable of reversible Mg‐ion insertion/extraction, the primary ions occupying the tetrahedral 8a sites and octahedral 16c sites along the Mg‐ion diffusion pathways are Zn and Mg. Although Zn ions could potentially act as factors hindering Mg‐ion diffusion, experimental observations have confirmed reversible cycling, suggesting that both Zn and Mg are mobile within the structure. In previous evaluations based on first‐principles calculations and the nudged elastic band (NEB) method for ZnCo_2_O_4_, the migration energies of Mg and Zn ions were found to be comparable. However, the NEB method is a technique for evaluating the hopping energy under limited local structural configurations, and it is challenging to assess the overall diffusivity of Mg and Zn under a wide variety of Zn/Mg/vacancy arrangements.

In this study, we applied a neural network potential (NNP) to the defect spinel structure Mg*_x_* ZnMnO_3_ with inserted Mg, and conducted a detailed computational analysis of the Mg insertion reaction and Mg^2^⁺ ion diffusivity. The NNP achieves computational accuracy comparable to conventional first‐principles calculations while enabling high‐speed simulations, thereby allowing large‐scale computational models to be studied within reasonable computational resources and time. Specifically, we performed detailed analyses focusing on the following three aspects:

1. Structural exploration of the most stable configurations and site occupancy analysis of Mg*_x_* ZnMnO_3_ at compositions *x* = 0, 0.16, 0.25, 0.33, 0.50, 0.68, 0.84, and 1.0 using a genetic algorithm [13].
2. Evaluation of thermodynamic stability and calculation of discharge voltages based on the obtained structures.
3. Evaluation of Mg^2^⁺ ion diffusion coefficients from molecular dynamics (MD) simulations, with comparative analysis against Zn and Mn ions.

Through these analyses, we elucidate the correlation between two‐phase coexistence regions and Mg^2^⁺ ion diffusivity in defect spinel materials and propose design guidelines for cathode materials for RMBs.

## 2 Methods

In this study, based on the previous work on Mg*_x_* Zn_0.94_Mn_1.03_O_3_ (0 < *x* < 1.03) [6], we investigated Mg*_x_* ZnMnO_3_ (*x* = 0, 0.16, 0.25, 0.33, 0.50, 0.68, 0.84, 1.0), in which cation vacancies were introduced at the 16d and 8a sites to maintain charge neutrality. First, the primitive unit cell of the defect spinel structure was expanded into a 3 × 3 × 3 supercell, and superstructure models containing up to 1440 + 288*x* atoms (Mg*_x_* ZnMnO_3_), including oxygen ions fixed at the 32e sites, were constructed. The corresponding compositions of the computational models are Mg*_x_*_’_Zn_288_Mn_288_O_864_, where *x*’ = 0, 48, 72, 96, 144, 196, 244, and 288.

Each cation site (8a, 16d, and 16c) was modeled to be occupiable by Mg, Zn, Mn, or vacancies, and all possible occupation patterns were considered in advance. For the interatomic interaction potential, we employed the pre‐trained neural network potential Preferred Potential (PFP) ver. 7.0 [14]. PFP ver. 7.0 is a graph neural network‐based potential trained on approximately 7 × 10⁷ first‐principles calculations covering 100 elements and has been reported to exhibit high reproducibility of first‐principles accuracy. In this study, this NNP was used to perform structural exploration of the most stable configurations by a genetic algorithm, structural relaxation, and molecular dynamics (MD) simulations in the NVT ensemble (1600 K, 1 ns, Δ*t* = 1 fs).

As a validation of the reliability of PFP, density functional theory (DFT) calculations were carried out for the Mg_3_Zn_10_Mn_11_O_32_ structure, and the predicted energies and forces obtained from the NNP were compared with those from DFT. The DFT calculations were performed using the Vienna Ab initio Simulation Package (VASP) [15], with a plane‐wave basis set and the projector augmented wave (PAW) method [16]. For the exchange–correlation functional, the generalized gradient approximation with PBEsol parameterization (GGA‐PBEsol) [17] was employed, combined with the GGA+U approach [18] to account for the on‐site Coulomb interactions in the Hubbard model. A *U* value of 3.9 eV was adopted for Mn. The plane‐wave cutoff energy was set to 500 eV.

The genetic algorithm (GA) was employed to optimize the configurations of Mg/Zn/Mn/vacancies, while oxygen atoms were fixed at the 32e sites. In the initial generation of chromosomes, random labels were assigned to each cation site. To generate offspring with more stable energy states, crossover and mutation operations were applied, and the most stable Mg/Zn/Mn/vacancy arrangements were explored. The evaluation of chromosomes was performed using the energies calculated by the NNP. Technical details of the genetic algorithm are described in our previous studies [19, 20, 21], and in the present work, we utilized our in‐house Python code “GmAte.py” [22].

## 3 Results and Discussion

### 3.1 Verification of NNP–DFT Accuracy

To quantitatively evaluate the reliability of the NNP employed in this study, DFT‐driven molecular dynamics (DFT‐MD) simulations were performed for Zn_10_Mn_11_O_32_, Mg_2_Zn_10_Mn_11_O_32_, Mg_3_Zn_10_Mn_11_O_32_, Mg_5_Zn_10_Mn_11_O_32_, and Mg_11_Zn_10_Mn_11_O_32_ at 1600 K for 2000 steps with a time step of Δ*t* = 1 fs. During the trajectories, total energies and atomic forces were computed for 2000 snapshots per composition. The same snapshots were then evaluated using the NNP to recalculate the energies and forces. Diagnostic plots of the results (Figure S1), comparing DFT (vertical axis) with NNP (horizontal axis), are listed in Table 1, and showed regression slopes close to unity (∼0.98–1.01) and coefficients of determination *R* ^2^ ≈ 0.98–0.99 for the energies, with energy RMSE in the range ∼1.8–4.3 meV atom^−1^. For the forces, slopes were likewise near unity (∼0.98–0.99) with *R* ^2^ ≈ 0.99 and an RMSE of ∼0.14 eV Å^−1^, demonstrating good accordance between DFT and NNP. These results confirm that the NNP used in this study retains first‐principles‐level accuracy across these representative compositions, thereby justifying its application in the energetics of Mg ion insertion reaction and ion dynamics analyses.

**TABLE 1:** Summary of diagnostic plots comparing DFT and NNP calculations for identical structures. For each composition, diagnostic plots were generated by comparing the energies and atomic forces obtained from DFT‐MD calculations (1600 K, 2000 steps) with the corresponding results from the NNP. The root mean square error (RMSE), coefficient of determination (*R* ^2^), and regression slope are listed. The diagnostic plots are provided in Figure S1.

Composition	Energy			Force		
	RMSE, meV atom^−1^	*R* ^2^	Reg. slope	RMSE, eV Å^−1^	*R* ^2^	Reg. slope
Zn_10_Mn_11_O_32_	4.3	0.98	0.98	0.14	0.99	0.98
Mg_2_Zn_10_Mn_11_O_32_	2.7	0.99	1.01	0.14	0.99	0.98
Mg_3_Zn_10_Mn_11_O_32_	2.8	0.99	0.98	0.14	0.99	0.99
Mg_5_Zn_10_Mn_11_O_32_	2.8	0.99	0.98	0.14	0.99	0.98
Mg_11_Zn_10_Mn_11_O_32_	1.8	0.99	1.01	0.14	0.99	0.98

### 3.2 Optimization of Cation Arrangements by GA

**FIGURE 1:**
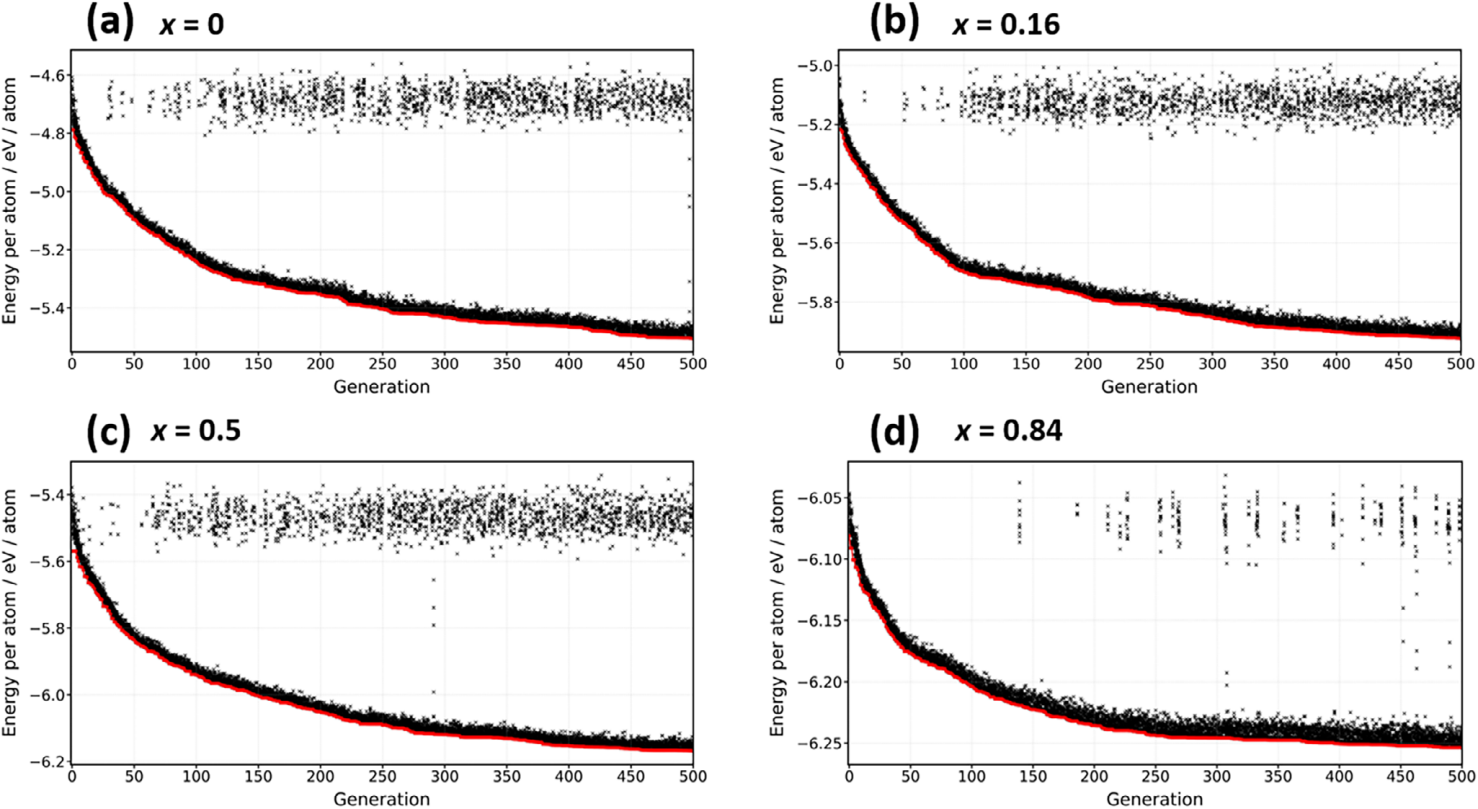
Convergence behavior of the GA structural optimization. The horizontal axis represents the generation number, and the vertical axis shows the energy of the obtained structures (eV atom^−1^). Panels (a)–(d) correspond to the results for compositions *x* = 0, 0.16, 0.50, and 0.84, respectively. The black crosses indicate the energies of individual structures in each generation, while the red points represent the energy of the most stable structure in the corresponding generation.

Figure 1 shows the energy convergence behavior of the GA for four representative compositions (*x* = 0.16, 0.25, 0.50, 0.84) out of the eight compositions investigated in this study. For all compositions, the energy difference relative to the previous generation of the most stable structure became less than 1 meV atom^−1^ within 500 generations. Although continuous structural stabilization was observed, the GA was terminated at 500 generations due to the small changes in energy values and considerations of computational resources.

The convergence plots for the remaining four compositions (*x* = 0, 0.33, 0.68, 1.00) exhibited similar behavior. The lowest‐energy structures obtained in the final generation were subsequently employed as input models for the following site occupancy analysis, formation energy calculations, and diffusion simulations.

### 3.3 Site Occupancy Tendencies

**FIGURE 2:**
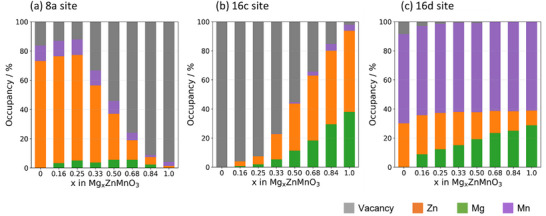
Composition‐dependent occupancies of each ion in (a) tetrahedral 8a sites, (b) octahedral 16c sites, and (c) octahedral 16d sites of the lowest‐energy structures obtained by GA for Mg*_x_* ZnMnO_3_ (0 ≤ *x* ≤ 1). In the 8a sites, Zn dominantly occupies the positions, but its occupancy gradually decreases beyond the stoichiometric spinel composition (*x* = 0.25). In contrast, the octahedral sites (16c and 16d) are primarily occupied by Mg and Mn, and the occupancy of Zn ions also increases with progressive Mg insertion. Ultimately, at *x* = 1.0, the tetrahedral sites become vacant, while the octahedral sites are nearly fully occupied by cations, completing the rock‐salt coordination.

Figure 2 presents stacked bar plots of the site occupancies for the lowest‐energy structures obtained by GA. The occupancies of Mg, Zn, Mn, and vacancies are shown as a function of Mg content *x* for each cation site: (a) tetrahedral 8a sites, (b) octahedral 16c sites, and (c) 16d sites.

First, considering the tetrahedral 8a sites (Figure 2a), at *x* = 0 the occupancies consist of approximately 75% Zn, 15% vacancies, and 10% Mn. As *x* increases, the Zn occupancy reaches a maximum at *x* = 0.25 and then begins to decrease. At *x* = 1.0, it falls below 5%, while the vacancy fraction rises to more than 90%. This indicates that Zn^2^⁺ strongly prefers the tetrahedral environment, whereas Mg^2^⁺ and Mn ions tend to occupy octahedral sites (16c or 16d). With Mg insertion, the occupancy of octahedral sites increases; in particular, when Mg ions occupy the 16c sites adjacent to the 8a sites, Zn ions originally located at the 8a sites are pushed into octahedral sites due to electrostatic repulsion between cations, leading to an increase in 8a‐site vacancies. The Mn occupancy at the 8a sites remains consistently low at around 5%–10%, demonstrating that Mn has a weaker preference for tetrahedral sites compared to Zn. In contrast, Mn ions exhibit a consistent tendency to occupy the 16d sites. Ultimately, at *x* = 1.0, all cations are accommodated in octahedral sites, forming a rock‐salt structure.

These results demonstrate that (i) Zn^2^⁺ preferentially occupies the tetrahedral 8a sites, (ii) Mn preferentially fills the octahedral 16d sites, (iii) Mg is inserted into both the 16c and 16d sites, and (iv) with increasing Mg content, Zn initially occupying the 8a sites is pushed into octahedral sites, leaving vacancies in the tetrahedral sites. These site occupancy tendencies are consistent with the trends observed in our previous GA calculations based on DFT [6].

### 3.4 Thermodynamic Stability and Voltage Evaluation

Thermodynamic stability was assessed using the thermodynamic decomposition energy (energy above hull, *E*_hull_) calculated from the composition dependence of the total energy [7]. When *E*_hull_ = 0, the phase is considered a stable phase (i.e., located on the convex hull and existing as a thermodynamically stable equilibrium phase at absolute zero), whereas increasingly positive values indicate reduced stability [23]. (It should be noted that the materials investigated in this study were limited to the spinel–rock‐salt type Mg*_x_* ZnMnO_3_ compositions. Therefore, the structures at *x* = 0 and 1 were evaluated as reference states with formation energy set to zero, and the calculated *E*_hull_ values do not represent those with respect to all possible Mg–Zn–Mn–O phases.)

**FIGURE 3:**
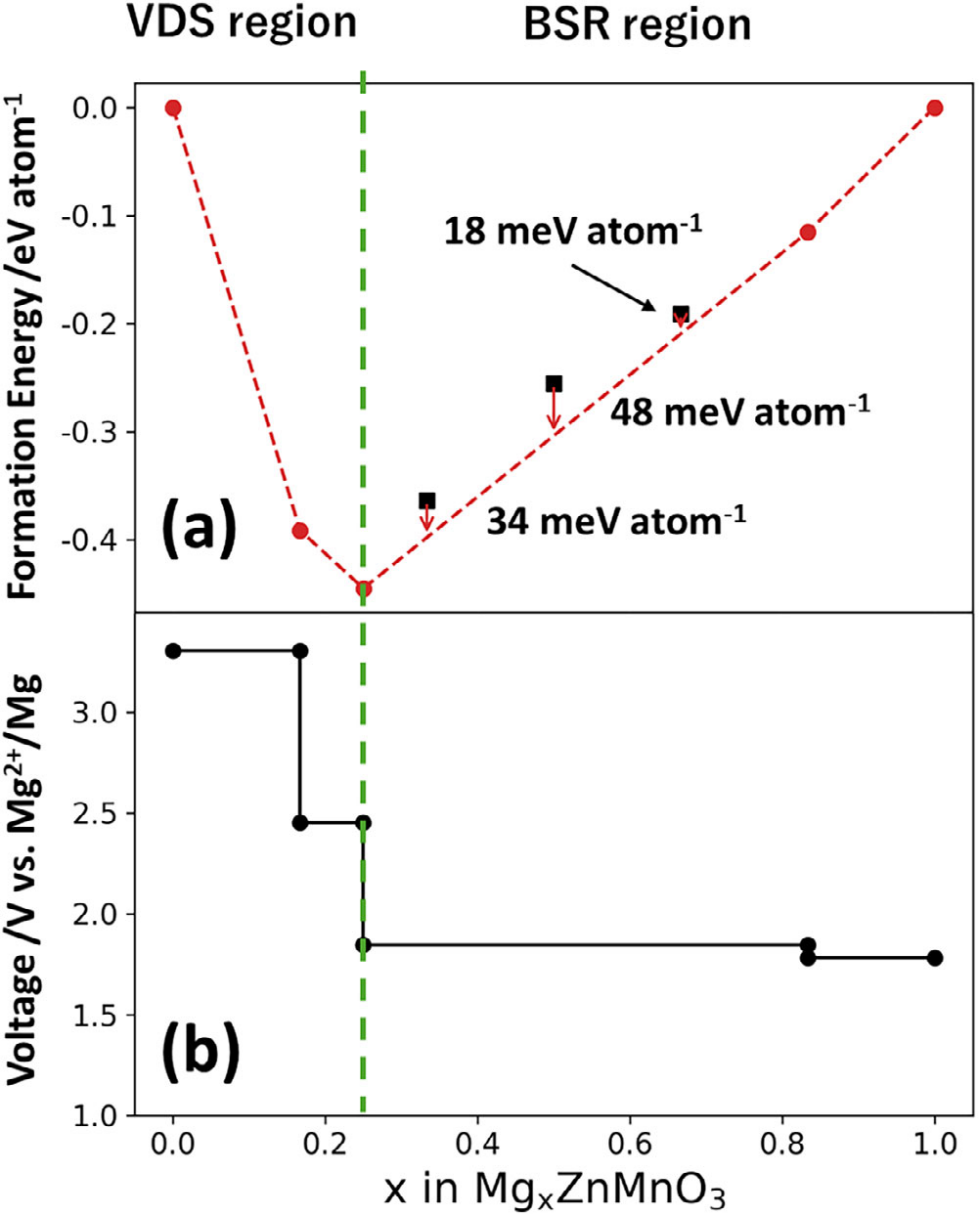
Composition dependence of the thermodynamic stability and average insertion voltage of Mg*_x_* ZnMnO_3_. (a) Formation energy plot of Mg*_x_* ZnMnO_3_ (0 ≤ *x* ≤ 1). For *x* ≤ 0.25, the reaction proceeds as a single‐phase reaction of cation‐deficient spinel, whereas for 0.25 < *x* < 0.84, spinel–rock‐salt biphasic coexistence is suggested. The composition *x* = 0.25 corresponds to the stoichiometric spinel (M_3_O_4_) and exhibits a minimum in energy. The composition ranges 0 < *x* < 0.25 and 0.25 < *x* < 1.0 are hereafter referred to as the vacancy‐driven spinel (VDS) region and the biphasic spinel–rock‐salt (BSR) region, respectively. (b) Average Mg insertion voltage (V vs. Mg^2^⁺/Mg) calculated from the slope of the convex hull in (a) (Δ*G*/*nF*). In the spinel solid‐solution region (*x* ≤ 0.25), a high voltage of ≈3.3 V is observed, while in the biphasic coexistence region the voltage gradually decreases to a plateau near ≈1.9–2.0 V. The green dashed line indicates the phase boundary at *x* = 0.25 between the spinel solid‐solution region (VDS) and the biphasic coexistence region (BSR).

As shown in the formation energy plot of Mg*_x_* ZnMnO_3_ (0 ≤ *x* ≤ 1) in Figure 3a, the formation energy reaches its minimum (and *E*_hull_ = 0) at *x* = 0.25. The composition *x* = 0.25 corresponds to the stoichiometric spinel ratio of cations to oxide anions (3:4), at which the occupancy of tetrahedral sites reaches its maximum. In the range 0 ≤ *x* ≤ 0.25, all investigated compositions lie on the convex hull, indicating that the Mg insertion/extraction proceeds as a single‐phase reaction via solid solution within the spinel framework. Since this region corresponds to cation‐deficient spinel structures, we hereafter refer to it as the vacancy‐driven spinel (VDS) region.

In contrast, for *x* > 0.25, the number of cations exceeds the stoichiometric spinel composition, and the excess cations occupy the vacant octahedral 16c sites. Consequently, the cation occupancy of the adjacent 8a sites tends to decrease due to electrostatic repulsion between cations (Figure 2). Therefore, in the composition range 0.25 ≤ *x* ≤ 1.00, a structural transformation from the spinel to the rock‐salt phase was observed with increasing *x*. Unlike the cation‐deficient spinel region (0 ≤ *x* ≤ 0.25), the compositions in the range 0.33 ≤ *x* ≤ 0.68 do not form a convex hull. Instead, the results suggest that in the range between *x* = 0.25 and *x* = 0.84, a biphasic coexistence reaction occurs between the spinel phase (Mg_0.25_ZnMnO_3_) and the rock‐salt phase (Mg_0.84_ZnMnO_3_). We, hereafter, refer to the composition range 0.25 ≤ *x* ≤ 1.00 as the biphasic spinel–rock‐salt (BSR) region.

It is noteworthy that the maximum *E*_hull_ value in this composition range is only 48 meV atom^−1^ (at *x* = 0.5). Considering that the energies obtained from NNP correspond to zero‐temperature energies, it is possible that the Mg insertion/extraction reactions proceed as solid‐solution reactions due to the effects of configurational entropy and finite‐temperature atomic vibrations. Alternatively, as proposed for Li insertion/extraction in olivine‐type LiFePO_4_, the reactions may also kinetically proceed via solid‐solution pathways [24].

Figure 3b shows the average (de)insertion voltage calculated from the same set of energies, revealing an operating voltage window of 1.8–3.3 V versus Mg/Mg^2^⁺. Within the spinel solid‐solution region (*x* ≤ 0.25), the voltage lies in the range of 2.5–3.3 V. In the biphasic coexistence region, a plateau appears near 1.9 V, while in the rock‐salt single‐phase region (*x* → 1), the voltage gradually decreases to 1.8–1.9 V. These behaviors are in good quantitative agreement with previous analyses based on first‐principles calculations, thereby supporting the validity of the models computed using the NNP.

Notably, the defect spinel region exhibits an operating voltage that is approximately 1.5 V higher than that of the biphasic coexistence region, indicating that the introduction of cation vacancies and the preservation of the spinel framework directly contribute to voltage enhancement. From the perspective of Mn oxidation states, Mn^3^⁺^/^⁴⁺ redox reactions are expected to occur in the range 0 ≤ *x* ≤ 0.50, whereas Mn^2^⁺^/^^3^⁺ redox reactions are likely to dominate in the range 0.50 ≤ *x* ≤ 1.0. Therefore, the observed changes in Mg insertion/extraction voltage are considered to originate not from the electronic states of Mn, but rather from the structural transformations of the crystal lattice.

### 3.5 Composition Dependence of Ion Diffusion Coefficients

**FIGURE 4:**
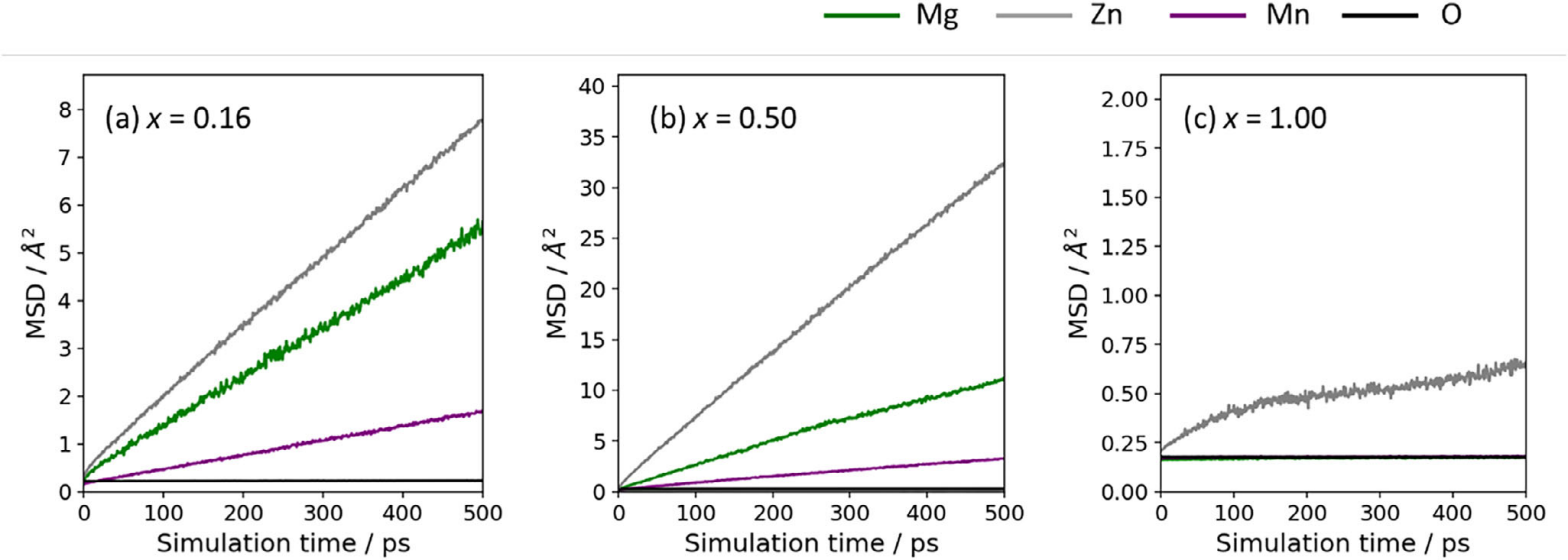
(a–c) MSD plots for representative compositions showing the composition dependence of ion diffusion coefficients. The NVT–MD simulations were performed at 1600 K with a production time of 1 ns and a time step of Δ*t* = 1 fs.

In this section, we present the results of evaluating the composition dependence of Mg‐ion diffusion based on the time evolution of the mean square displacement (MSD) obtained from MD simulations performed in the NVT ensemble (1600 K, 1 ns, Δ*t* = 1 fs). The simulation temperature was chosen such that the number of hopping events of multivalent Mg ions would be statistically sufficient within the 1 ns simulation time. As will be discussed later, no oxygen diffusion was observed in any of the compositions, confirming that the oxygen framework remained intact even under high‐temperature conditions. Figure 4a–c shows the MSD curves for representative compositions: *x* = 0.16 (VDS region), 0.50 (BSR region), and 1.0 (rock‐salt structure). For each ionic species, the MSD–time curves were linearly fitted in the interval of 200–500 ps by the least‐squares method, and the diffusion coefficient *D* was calculated from the slope. In addition, for compositions corresponding to the biphasic coexistence region, diffusion coefficients were also evaluated from MSD to discuss the fundamental behavior of Mg‐ion conduction.

As shown in Figures S2 and S3, the potential energy becomes constant after the initial ∼10 ps of the MD run. To ensure that only equilibrated data were used, we discarded the first 200 ps of each trajectory and performed the MSD analysis on the remaining portion.

For *x* = 0.16 in the defect spinel region (Figure 4a), the MSD of Mg exhibits good linearity with respect to time, clearly indicating diffusive behavior (*D*_Mg_ = 1.73 × 10^−7^ cm^2^ s^−1^). In contrast, at *x* = 0.50 (Figure 4b), the slope further increases, yielding the maximum value of *D*_Mg_ = 3.97 × 10^−7^ cm^2^ s^−1^. This result suggests that in the compositional domain where the spinel and rock‐salt frameworks coexist, fluctuations in cation arrangements and vacancy distributions create the most favorable conditions for opening diffusion pathways. While Mg diffusion is sufficiently observed even in the defect spinel region (*x* < 0.25), its value further increases upon entering the biphasic coexistence region, which is a characteristic feature of this system.

Moreover, for both *x* = 0.16 and 0.50, diffusion behavior of Zn and Mn ions was also observed. In particular, Zn, which is also a divalent ion, exhibited larger MSD slopes than Mg and thus serves as the primary diffusing species.

In contrast, for the stoichiometric spinel composition (cation‐to‐anion ratio = 3:4) at *x* = 0.25 (not shown) and the stoichiometric rock‐salt composition (cation‐to‐anion ratio = 1:1) at *x* = 1.0 (Figure 4c), the NNP‐MD results show that the increase in MSD is extremely slow, with the MSD values not exceeding 3 Å^2^ even after 1 ns (*D*_Mg_ = 6.56 × 10^−10^ cm^2^ s^−1^).

**FIGURE 5:**
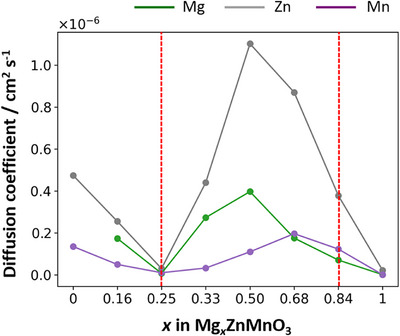
Composition dependence of the diffusion coefficients of each cation species (Mg, Zn, Mn) at 1600 K. The region enclosed by the red dashed lines corresponds to the biphasic coexistence region (0.25 < *x* < 0.84, Figure 3) suggested from the GA‐optimized structures.

The diffusion coefficients derived from the above MSD analyses for each composition are plotted in Figure 5. The Mg‐ion diffusion behavior is found to be strongly dependent on composition, with significantly higher diffusivity observed in the cation‐excess spinel to rock‐salt biphasic coexistence region compared to the cation‐deficient spinel region. Furthermore, at the stoichiometric spinel composition (*x* = 0.25) and the stoichiometric rock‐salt composition (*x* = 1.0), cation diffusion is essentially frozen relative to other compositions. These results clearly indicate that ion diffusion is driven by defects in the structure.

**FIGURE 6:**
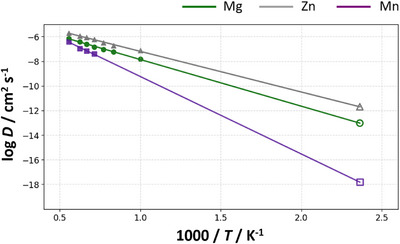
Arrhenius plots of cation diffusivity for Mg (green plots), Zn (gray plots), and Mn (purple plots) in Mg_0.50_ZnMnO_3_. The extracted activation energies are 0.75 eV for Mg, 0.66 eV for Zn, and 1.70 eV for Mn, and the diffusivity ranking Zn > Mg > Mn is preserved over the entire temperature range. The Arrhenius behavior remains linear down to 1000 K, indicating that the trends can be extrapolated toward the practical operating regime (∼150°C) and are, therefore, relevant to experimental conditions.

To relate high‐*T* diffusion data to device‐relevant conditions, we further performed MD simulations for Mg_0.50_ZnMnO_3_ at seven temperatures, *T* = 1000, 1200, 1300, 1400, 1500, 1600, 1800 K. Activation energies were obtained from linear fits of log_10_ *D* versus 1000/*T* (Arrhenius plots in Figure 6). Due to small MSD change for Mn ions less than 1400 K, we evaluated activation energies using the temperature range from 1400 to 1800 K. The fits are linear across the probed range, yielding activation energies of 0.75 eV (Mg), 0.66 eV (Zn), and 1.70 eV (Mn). The diffusivity ranking Zn > Mg > Mn is preserved at all temperatures. Because the Arrhenius behavior remains linear down to 1000 K for Mg and Zn and oxygen frame is maintained, these trends project smoothly toward the practical operating regime (∼ 430 K) [6].

However, given the large temperature gap between 1000 and 430 K, deviations between extrapolated and experimentally observed diffusivities may arise due to factors such as volume changes or order–disorder transitions of Mg/Zn/vacancies. Nevertheless, because charge–discharge behavior has been experimentally observed for Mg‐ion batteries, it is highly likely that Zn—which exhibits higher mobility than Mg—also participates in simultaneous Mg/Zn diffusion near ∼430 K.

### 3.6 Visualization of Diffusion Pathways

**FIGURE 7:**
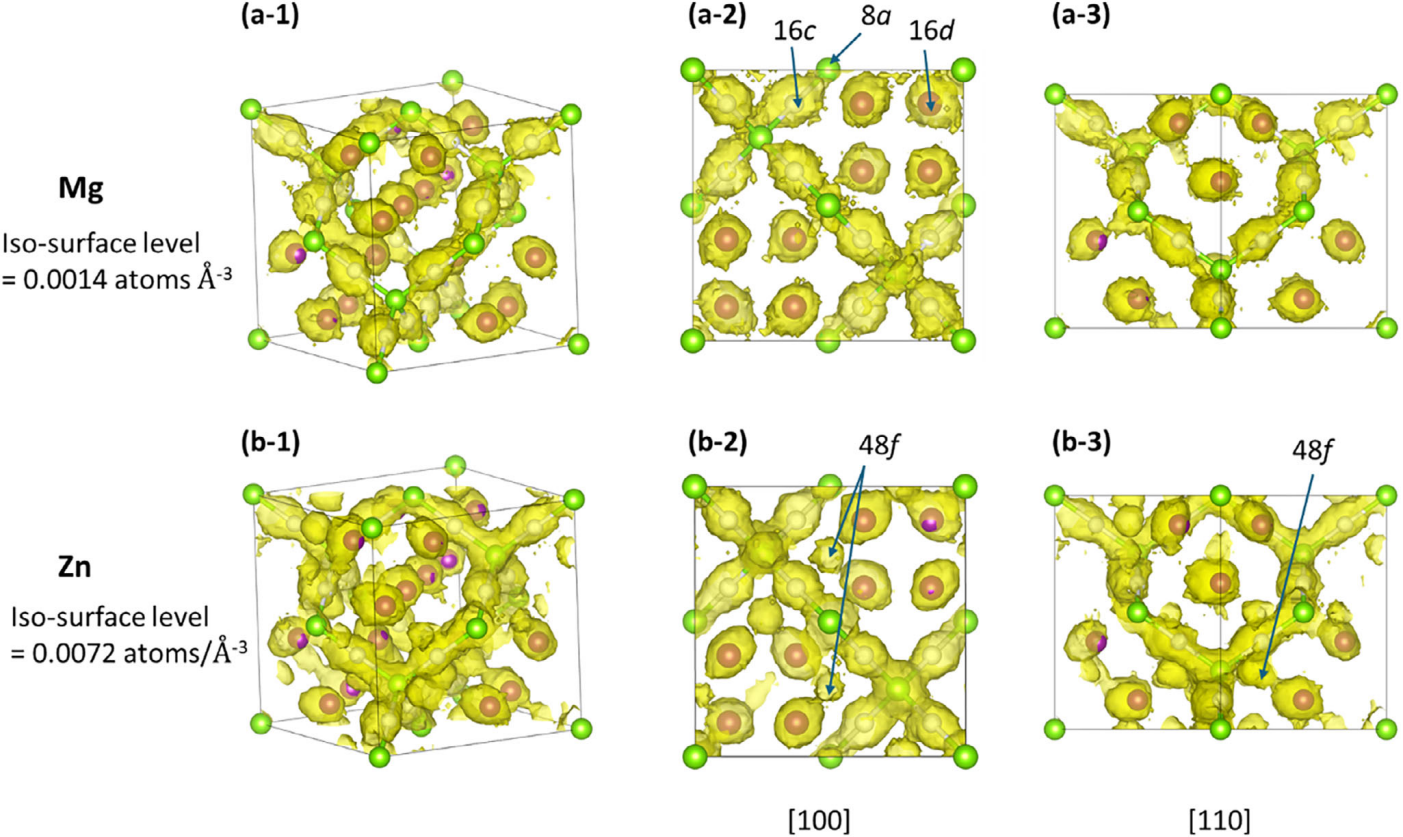
Diffusion pathways of Mg and Zn in Mg_0.50_ZnMnO_3_ (*x* = 0.50) obtained from NVT–MD simulations (1600 K, 1 ns, Δ*t* = 1 fs). (a) Mg and (b) Zn population density distributions are shown as isosurfaces from three different perspectives. The middle (−2) and right (−3) panels correspond to projections along the [100] and [110] directions, respectively. The iso‐surface levels for Mg and Zn are set to 0.0014 and 0.0072 atoms Å^−3^, respectively. Green, white, and purple spheres indicate the 8a, 16c, and 16d sites, respectively (assuming *Fd3m* symmetry). Both species exhibit high probability densities along the characteristic spinel diffusion channel (8a → 16c → 8a), indicating that Mg and Zn migrate along the same topological pathways. In contrast, the 16d sites tend to remain isolated; however, weak population densities observed at the 48f positions suggest that ions may occasionally hop to adjacent 16c or 16d sites via vacant 48f tetrahedral sites.

To investigate the ion conduction mechanism further, we analyzed the representative composition *x* = 0.50 (Mg_0.50_ZnMnO_3_) within the biphasic coexistence region (BSR region), where Mg exhibits the maximum diffusion coefficient. From the MD simulation trajectory data, the population densities of Mg and Zn were calculated, and the diffusion pathways of each ion were three‐dimensionally visualized (Figure 7). For visualization, the 3 × 3 × 3 superstructure model was reduced back to the size of the original lattice, and the population densities were superimposed.

From Figure 7, it was confirmed that both Mg and Zn form dense probability distributions along the typical spinel diffusion channels of 8a → 16c → 8a [25]. In contrast, Mg and Zn ions located at the 16d sites tend to remain isolated and are considered to contribute little to diffusion (although some ions were observed to hop from the 16d site to a 16c or 16d site via the tetrahedral vacant 48f site, and sparse population densities were suggested by adjusting the isosurface levels. In fact, part of the Zn population density indicates diffusion pathways mediated through the 48f sites). These results indicate that Mg and Zn essentially migrate by sharing the same crystallographic topological pathways.

This finding suggests two possible implications: on one hand, the presence of Zn ions along the diffusion pathway could hinder Mg‐ion diffusion; on the other hand, if Zn ions possess higher diffusivity, they may assist Mg‐ion transport via a concerted hopping mechanism. Here, “concerted” refers to the possibility that when one Mg (or Zn) ion migrates between sites, neighboring cations (particularly interstitial Mg) or vacancies are temporally rearranged in close proximity, triggering a chain‐like hopping process similar to an interstitialcy mechanism. Especially in systems with *x* > 0.25, adjacent 8a and 16c sites can both be occupied by Mg or Zn ions, and strong electrostatic repulsions between them may facilitate hopping. Indeed, in the present simulations, the diffusion coefficient of Zn ions was larger than that of Mg ions, suggesting that Zn ions may have promoted Mg‐ion diffusion.

**FIGURE 8:**
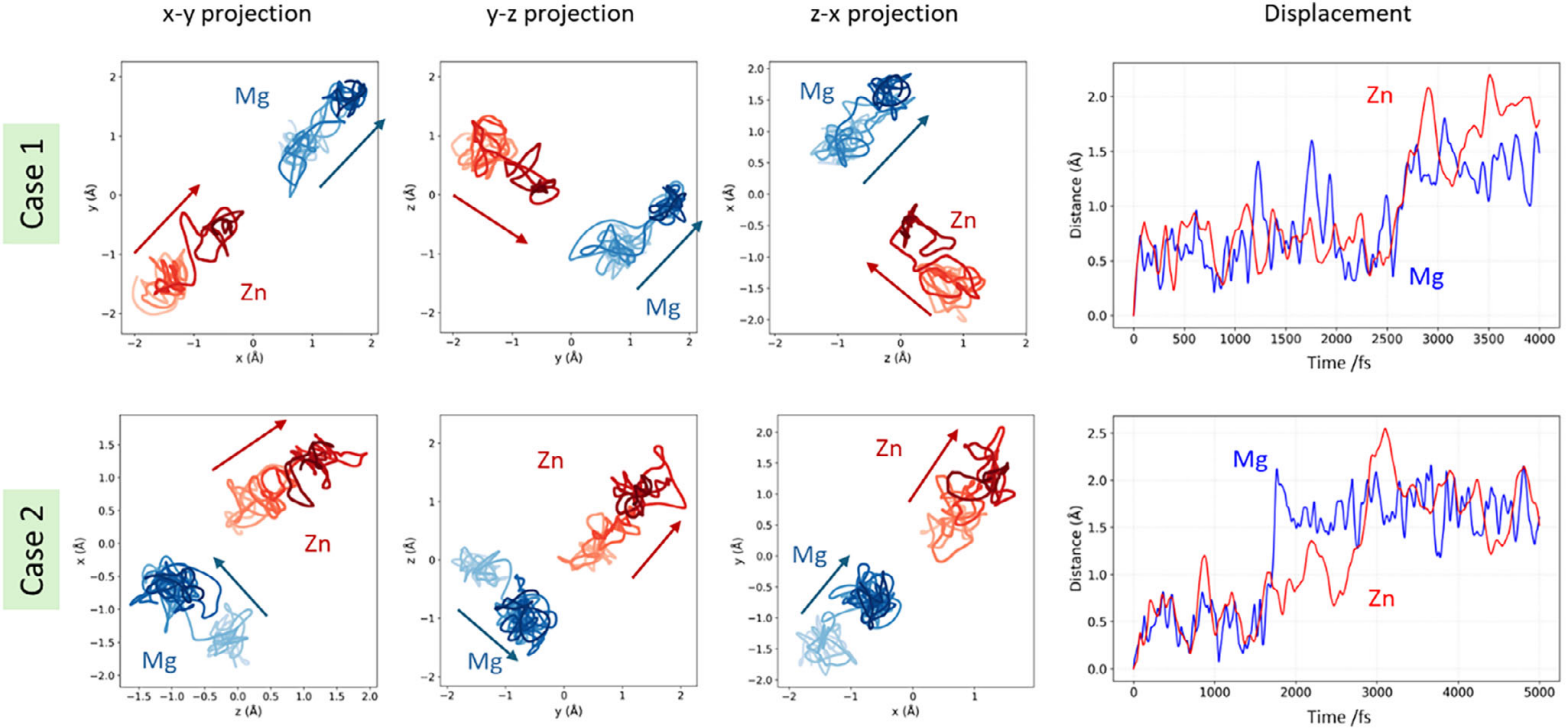
Two examples of concerted hopping events, in which Mg and Zn ions hop simultaneously, extracted from the NNP‐MD trajectories at 1600 K for 1 ns with a time step of 1 fs. The panels in the upper and lower rows correspond to Case 1 and Case 2, respect for y. The three panels on the left show the trajectories of the selected Mg (blue) and Zn (red) ions projected onto the *x*–*y*, *y*–*z*, and *z*–*x* planes. The color changes from light to dark as time progresses, and the arrows indicate the overall direction of the hopping. The rightmost panel shows the displacements: the magnitude of the displacement of the selected Mg (blue) and Zn (red) ions from an arbitrarily chosen initial time *t* = 0 (vertical axis, in Å) is plotted as a function of time (horizontal axis, in fs). In Case 1, an increase in displacement attributable to hopping is observed around 2500–3000 fs, whereas in Case 2 it is observed around 1800–3000 fs.

Figure 8 shows two representative cases, manually extracted from the NNP‐MD trajectories at the composition *x* = 0.5, where the diffusion coefficient is the highest, in which neighboring Mg and Zn ions separated by about 3 Å undergo concerted hopping. In Case 1, both the Mg and Zn ions initially located at tetrahedral sites hop almost simultaneously into neighboring octahedral sites. In contrast, in Case 2 the Mg ion initially occupying an octahedral site first hops into a tetrahedral site, and after a time lag of about 1 ps the Zn ion hops from a tetrahedral site to an octahedral site. This concerted hopping behavior of the divalent ions is partially consistent with the latter hypothesis discussed above; however, since it is based on only two observed events, it is difficult to draw a firm conclusion at this stage, and further quantitative analysis will be required in future work.

### 3.7 Discussion

As shown in Figure 5, the diffusion coefficients of Mg, Zn, and Mn in Mg*_x_* ZnMnO_3_ (0 ≤ *x* ≤ 1) are strongly dependent on composition, exhibiting two distinctly different behaviors across separate compositional regions. Here, the relative mobilities of the cations are compared with respect to Mg, and the trends are summarized from the perspective of diffusion pathway formation.

First, the diffusion coefficients are drastically suppressed at the stoichiometric spinel composition *x* = 0.25 and the rock‐salt structure at *x* = 1.0. In these compositions, cation vacancies and interstitial cations are nearly eliminated, leading to suppressed diffusion of both Mg and Zn. However, since this phenomenon occurs only at these limited compositions, its influence on the electrochemical Mg‐ion charge/discharge process, which is expected to proceed with continuous compositional changes, is considered to be limited.

In contrast, (i) the VDS region at *x* < 0.25 and (ii) the BSR region at 0.25 < *x* < 0.84 exhibit significantly enhanced diffusion coefficients for Mg and Zn. In the former, hopping pathways mediated by vacancies dominate, and hopping can occur with relatively low barriers as long as the vacancies are interconnected. In the latter, the introduction of excess Mg is expected to create situations in which adjacent tetrahedral 8a and octahedral 16c sites are occupied by Mg or Zn ions, potentially giving rise to concerted hopping [26].

Therefore, from the perspective of Mg‐ion diffusivity, it can be considered that relatively high diffusivity can be maintained across a wide compositional range of 0 ≤ *x* ≤ 1. However, from the viewpoint of phase stability as shown in Figure 3, the compositional range of 0.25 ≤ *x* ≤ 0.84 corresponds to a biphasic coexistence, and thus thermodynamically the system should follow the relatively low Mg‐ion diffusivities of the stoichiometric compositions at *x* = 0.25 and *x* = 0.84. Indeed, in Mg*_x_* Co_2_O_4_ spinel materials, it has been reported that upon Mg insertion, the particle surface is covered by a rock‐salt Mg_2_Co_2_O_4_ phase with extremely low Mg‐ion diffusivity, which hinders ion transport into the bulk and results in reduced capacity [27]. In the present ZnMnO_3_ material, the *E*_hull_ value in the BSR region (0.25 < *x* < 1.0) is as low as 48 meV atom^−1^, suggesting that, as proposed previously for Li*_x_* FePO_4_ materials [24], Mg‐ion insertion/extraction may kinetically proceed via a solid‐solution reaction. Importantly, the diffusion coefficients reported in Section 3.5 were obtained from homogeneous, single‐phase supercells, and thus, characterize transport within a metastable solid solution rather than the equilibrium two‐phase mixture. At the representative intermediate composition *x* = 0.50, the reported diffusivity corresponds to the metastable single‐phase *x* = 0.50 solid solution. We computed the overpotential required to stabilize this *x* = 0.50 solid solution against the equilibrium tie‐line between *x* = 0.25 and *x* = 0.84 and obtained a small value of ∼0.09 V. Together with the low *E*_hull_ in this region and the higher Mg diffusivity at *x* = 0.50 relative to the stoichiometric endpoints, these considerations suggest that transport via the metastable *x* = 0.50 solid solution could be favored over the equilibrium two‐phase pathway, since phase separation is kinetically hindered. In other words, Mg insertion reaction proceeds through a solid‐solution pathway.

Even if a biphasic coexistence reaction occurs, ensuring that neither of the two phases corresponds to a stoichiometric composition would allow Mg‐ion diffusion pathways to be maintained, even if a Mg‐rich shell phase forms on the particle surface. Indeed, in the present calculations, the results in Figure 3 suggest the emergence of a Mg_0.84_ZnMnO_3_ phase in the biphasic coexistence reaction. Thus, even if a Mg‐rich phase forms at the particle surface, Mg ions are expected to be able to diffuse through the shell phase.

Based on these findings, it is suggested that material design strategies such as multi‐element doping to introduce high configurational entropy could be effective for promoting solid‐solution reactions thermodynamically in ZnMnO_3_. Accordingly, the following design strategies for spinel‐type oxide materials as RMB cathodes are proposed.

- Promotion of spinel structural stabilization through the incorporation of tetrahedral site–preferring Zn ions.
- Potential Zn assistance to Mg‐ion diffusion through occasional concerted‐like events.
- Suppression of biphasic coexistence reactions involving stoichiometric compositions by the introduction of defects.

## 4 Conclusion

In this study, we investigated the Mg insertion system Mg*_x_* ZnMnO_3_ (0 ≤ *x* ≤ 1), derived from the defect spinel ZnMnO_3_, by integrating structural exploration of the most stable configurations using a genetic algorithm (GA) with neural network potentials (NNP), thermodynamic convex hull analysis and average voltage evaluation, and diffusion coefficient and pathway analysis based on molecular dynamics (MD). As a result, we proposed useful guidelines for the design of cathode materials for rechargeable Mg batteries.

From the analysis of the cation configurations obtained by GA, it was shown that Zn preferentially occupies tetrahedral 8a sites, whereas Mg tends to occupy octahedral 16d and/or 16c sites. NNP‐MD simulations at 1600 K revealed that the diffusion coefficients are strongly dependent on composition *x*. At the stoichiometric spinel composition (*x* = 0.25) and the stoichiometric rock‐salt composition (*x* = 1.0), diffusion becomes extremely small, whereas in the VDS region (*x* < 0.25) and the BSR region (0.25 < *x* < 1.0), appreciable diffusion coefficients were observed. These results indicate that Mg‐ion diffusion is enabled by the introduction of defects leading to non‐stoichiometry. In addition, Zn‐ion diffusion was also observed, and Zn was found to consistently exhibit higher diffusion coefficients than Mg.

Population density analysis supports that both Mg and Zn utilize the common 8a–16c–8a network. This observation, together with their distinct site preferences (Zn: tetrahedral; Mg: octahedral), is compatible with occasional concerted‐like rearrangements in which local electrostatic interactions can facilitate hopping as indicated in Figure 8. However, a quantitative analysis of the concerted hopping events has not yet been achieved, and further evaluation will be required.

Although biphasic coexistence reactions are expected to occur in the region *x* > 0.25, the formation of stoichiometric phases (*x* = 0.25, 1.0) would result in the precipitation of low‐diffusivity phases at particle surfaces, leading to capacity degradation. However, Mg*_x_* ZnMnO_3_ exhibits very low *E*_hull_ values, suggesting the possibility that the reactions proceed as solid‐solution reactions. Moreover, it was proposed that controlling configurational entropy by multi‐element doping to promote solid‐solution behavior throughout the entire composition range would be an important future material design strategy.

## Conflicts of Interest

The authors declare no conflicts of interest.

## Supporting information




**Supporting File**: asia70514‐sup‐0001‐SuppMat.docx

## Data Availability

The data that support the findings of this study are available from the corresponding author upon reasonable request.
