# Supplementary material for: Zn‐Assisted Mg Ion Transport in Spinel Oxide Cathodes: Insights From Neural Network Simulations
Source: Chem Asian J. 2025 Dec 15;21(2):e00908. doi: 10.1002/asia.202500908 (PMC12812791; doi:10.1002/asia.202500908)
Supplement: Supplementary file 1 — Supporting File: asia70514‐sup‐0001‐SuppMat.docx [file ASIA-21-e00908-s001.docx]

**Supporting Information for Publication**

Zn-Assisted Mg Ion Transport in Spinel Oxide Cathodes: Insights from Neural Network Simulations

Riku Nakahara^1^, Naoto Tanibata^1^, Hayami Takeda^1^, Masanobu Nakayama^1^*, Kohei Shimokawa^2,3^, Tetsu Ichitsubo^3^

1. Department of Materials Science and Engineering, Nagoya Institute of Technology, Gokiso-cho, Showa-ku, Nagoya, 466-8555 Japan
2. Frontier Research Institute for Interdisciplinary Sciences, Tohoku University, 2-1-1 Katahira, Aoba-ku, Sendai, 980-8577 Japan
3. Institute for Materials Research, Tohoku University, 2-1-1 Katahira, Aoba-ku, Sendai, 980-8577 Japan


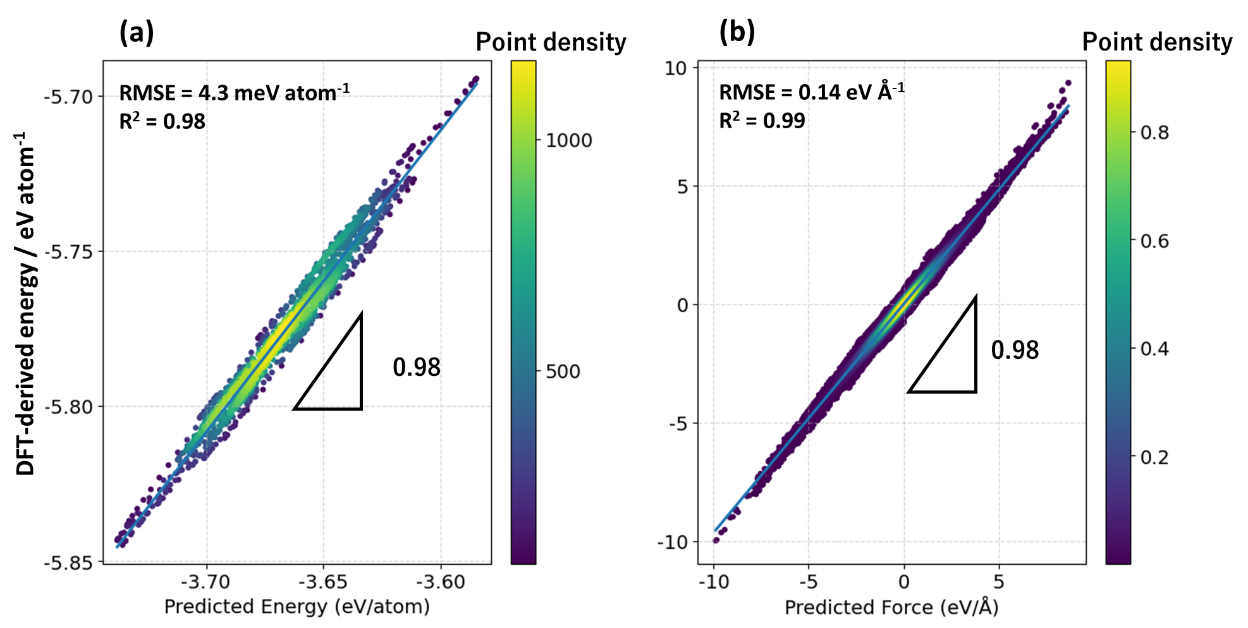


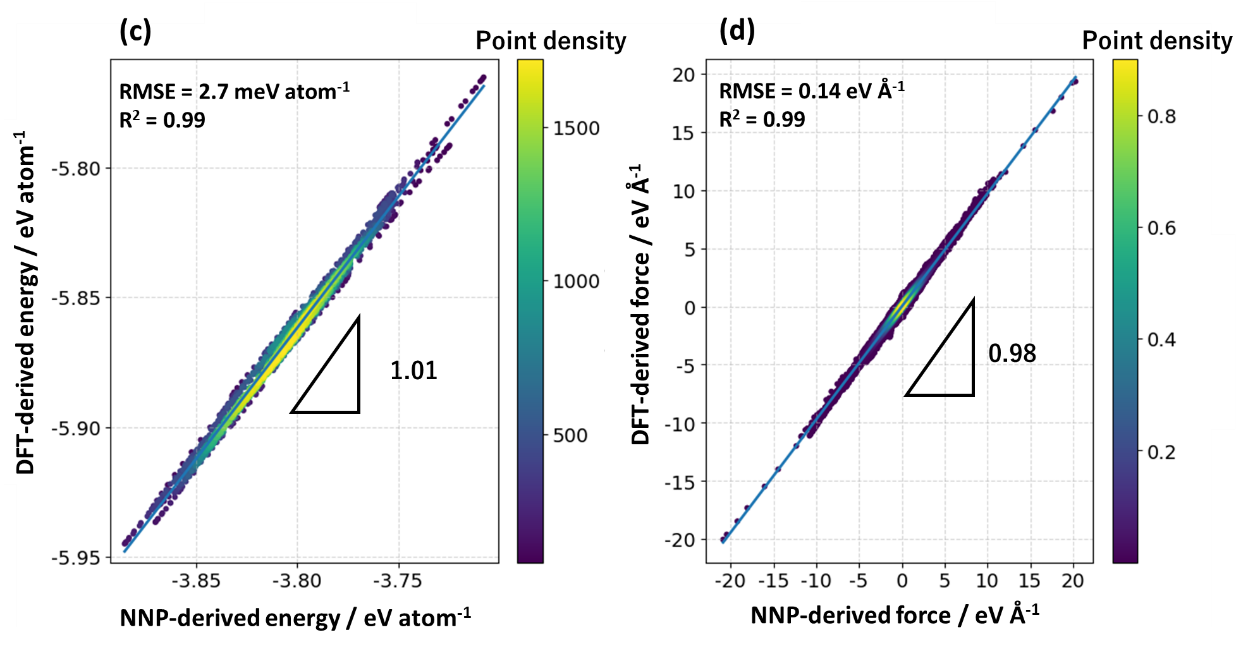


**Figure S1** Example of diagnostic plots comparing NNP and DFT-MD calculations for Zn_10_Mn_11_O_32_, Mg_2_Zn_10_Mn_11_O_32_, Mg_3_Zn_10_Mn_11_O_32_, Mg_5_Zn_10_Mn_11_O_32_, and Mg_11_Zn_10_Mn_11_O_32_ at 1600 K over 2 ps. Panels (a–j) correspond to diagnostic plots of the total energy (left) and atomic forces (right) for each composition: (a, b) Zn_10_Mn_11_O_32_, (c, d) Mg_2_Zn_10_Mn_11_O_32_, (e, f) Mg_3_Zn_10_Mn_11_O_32_, (g, h) Mg_5_Zn_10_Mn_11_O_32_, (i, j) Mg_11_Zn_10_Mn_11_O_32_. In all cases, both the regression slope and the coefficient of determination (R²) are close to unity, demonstrating that the NNP accurately reproduces the DFT-level energetics and forces across the different Mg contents.


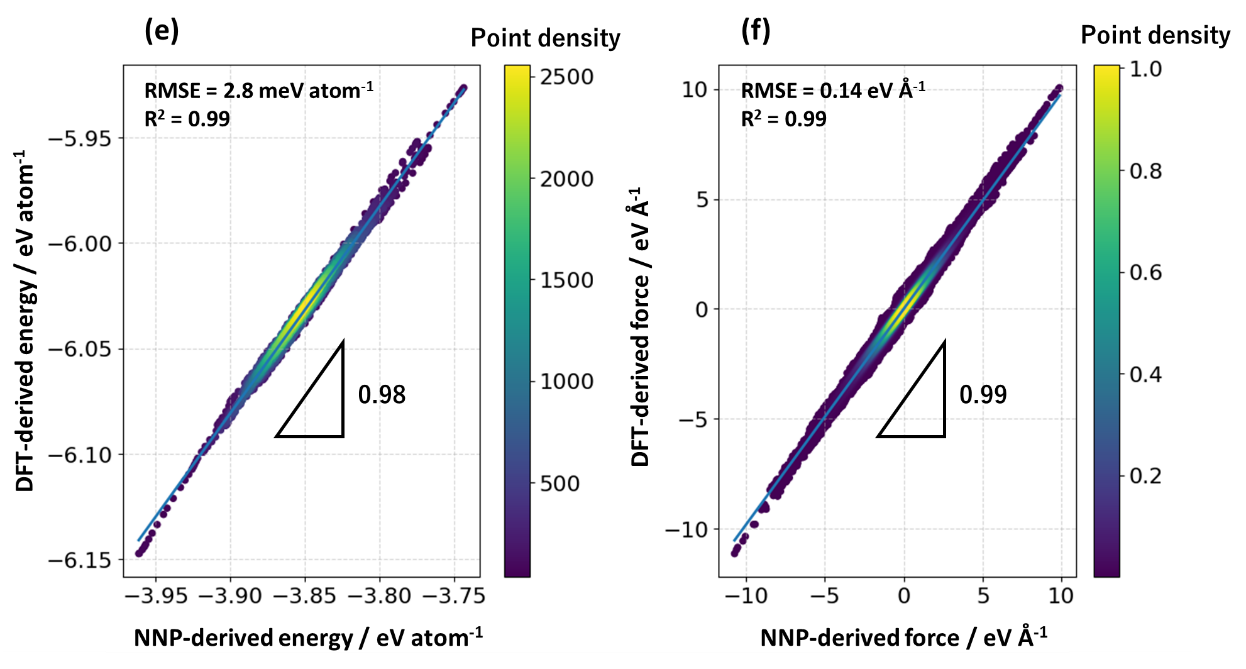


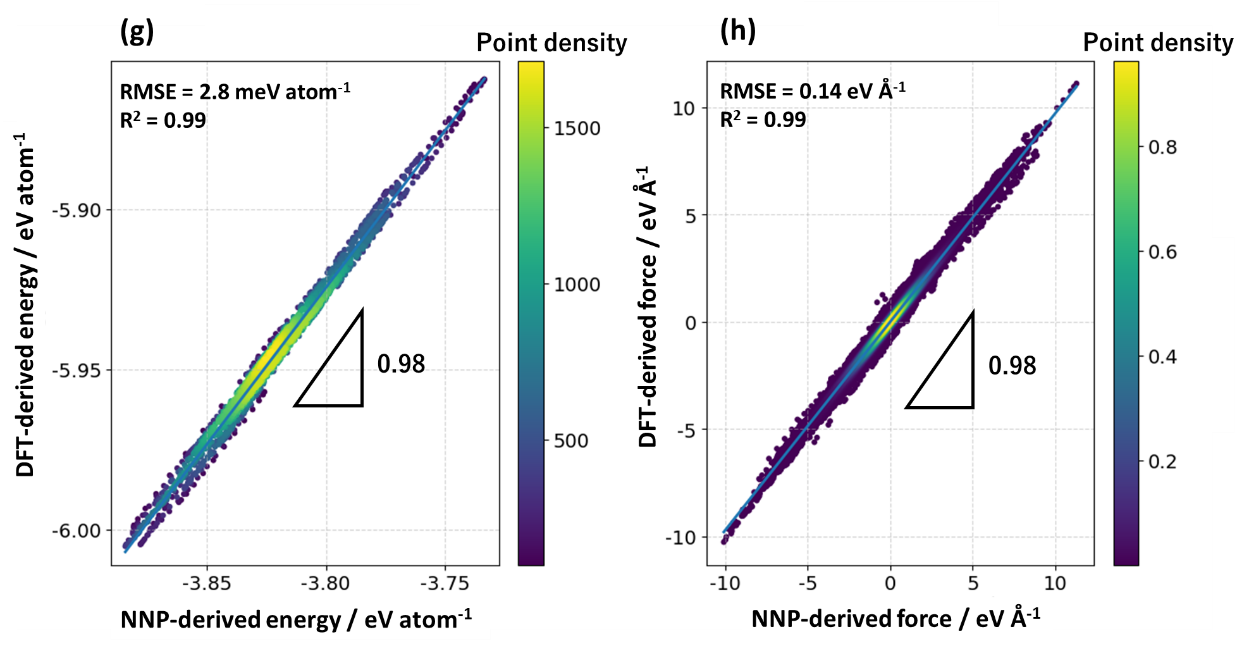


**Figure S1 (continued)** Example of diagnostic plots comparing NNP and DFT-MD calculations for Zn_10_Mn_11_O_32_, Mg_2_Zn_10_Mn_11_O_32_, Mg_3_Zn_10_Mn_11_O_32_, Mg_5_Zn_10_Mn_11_O_32_, and Mg_11_Zn_10_Mn_11_O_32_ at 1600 K over 2 ps. Panels (a–j) correspond to diagnostic plots of the total energy (left) and atomic forces (right) for each composition: (a, b) Zn_10_Mn_11_O_32_, (c, d) Mg_2_Zn_10_Mn_11_O_32_, (e, f) Mg_3_Zn_10_Mn_11_O_32_, (g, h) Mg_5_Zn_10_Mn_11_O_32_, (i, j) Mg_11_Zn_10_Mn_11_O_32_.In all cases, both the regression slope and the coefficient of determination (R²) are close to unity, demonstrating that the NNP accurately reproduces the DFT-level energetics and forces across the different Mg contents.


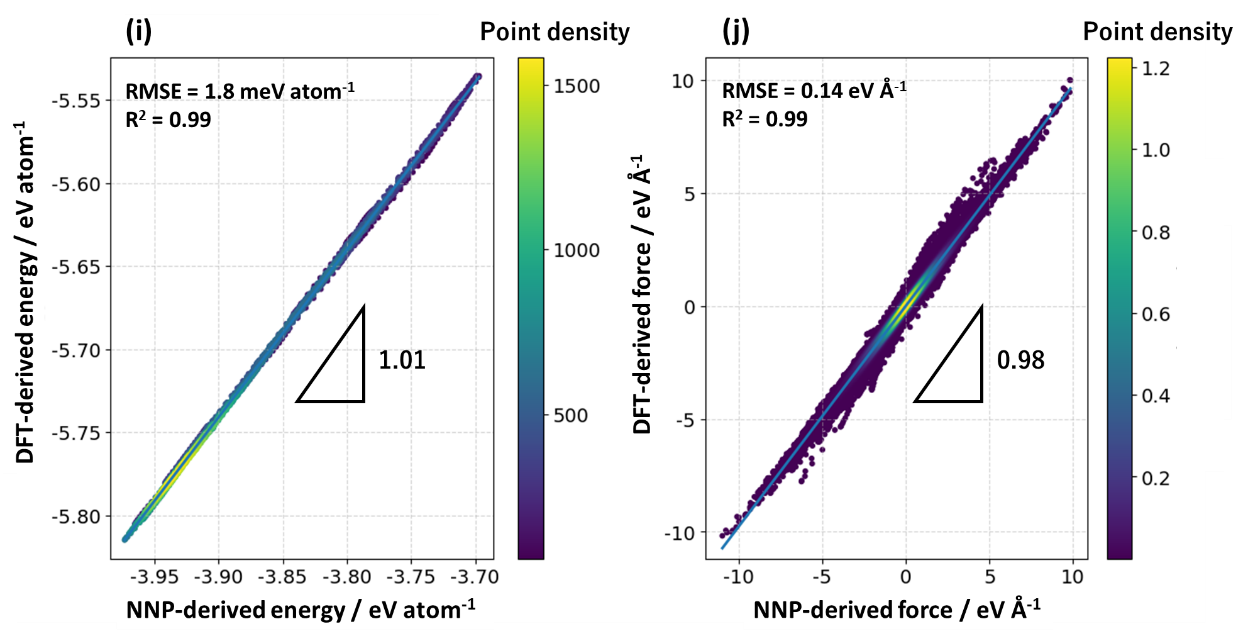


**Figure S1 (continued)** Example of diagnostic plots comparing NNP and DFT-MD calculations for Zn_10_Mn_11_O_32_, Mg_2_Zn_10_Mn_11_O_32_, Mg_3_Zn_10_Mn_11_O_32_, Mg_5_Zn_10_Mn_11_O_32_, and Mg_11_Zn_10_Mn_11_O_32_ at 1600 K over 2 ps. Panels (a–j) correspond to diagnostic plots of the total energy (left) and atomic forces (right) for each composition: (a, b) Zn_10_Mn_11_O_32_, (c, d) Mg_2_Zn_10_Mn_11_O_32_, (e, f) Mg_3_Zn_10_Mn_11_O_32_, (g, h) Mg_5_Zn_10_Mn_11_O_32_, (i, j) Mg_11_Zn_10_Mn_11_O_32_.In all cases, both the regression slope and the coefficient of determination (R²) are close to unity, demonstrating that the NNP accurately reproduces the DFT-level energetics and forces across the different Mg contents.


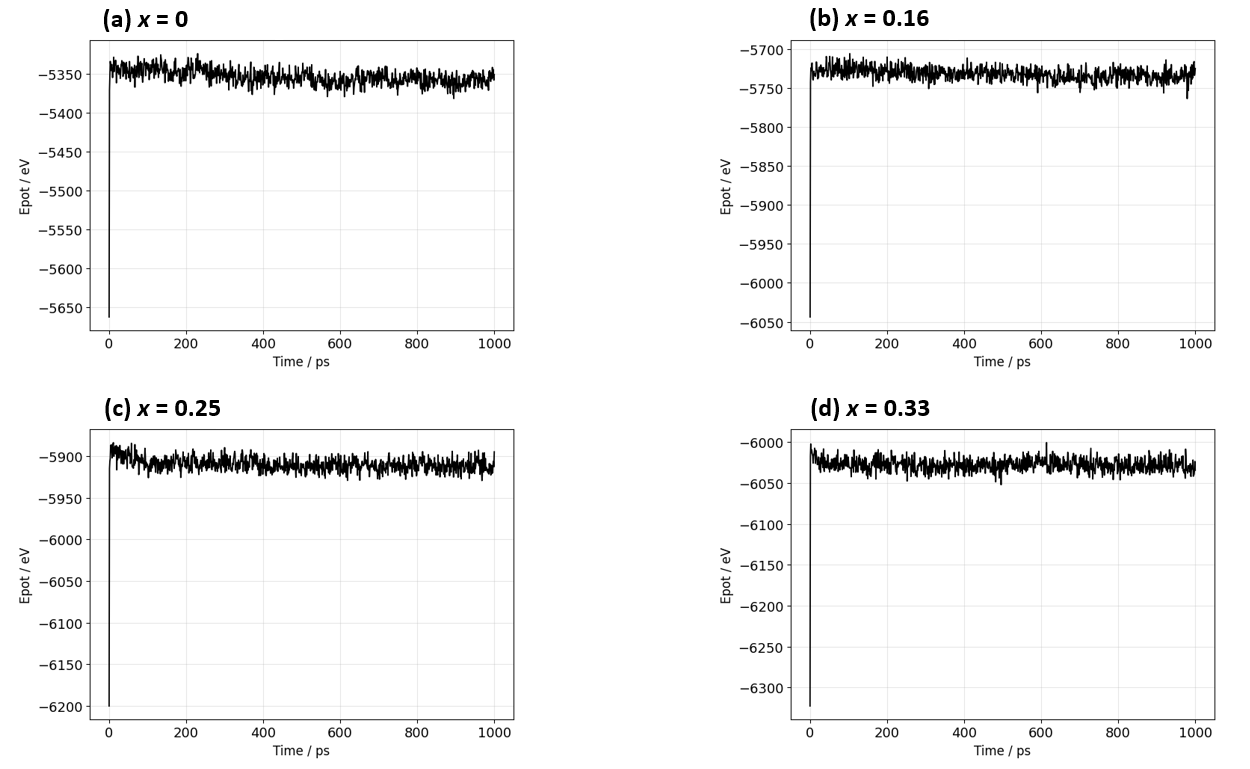


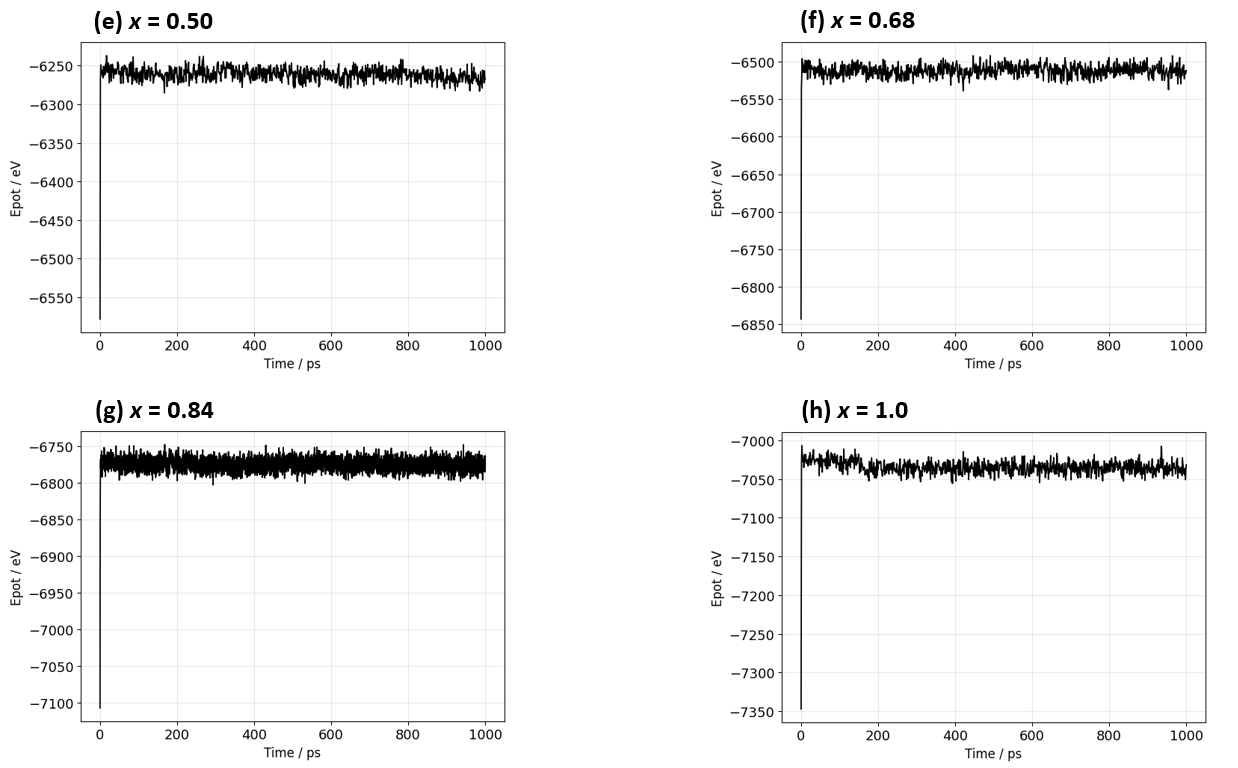


**Figure S2.** Potential energy profile as a function of MD time (0–10 ps) at 1600 K for various compositions, *x* = 0, 0.16, 0.25, 0.33, 0.50, 0.68, 0.84, and 1.0.


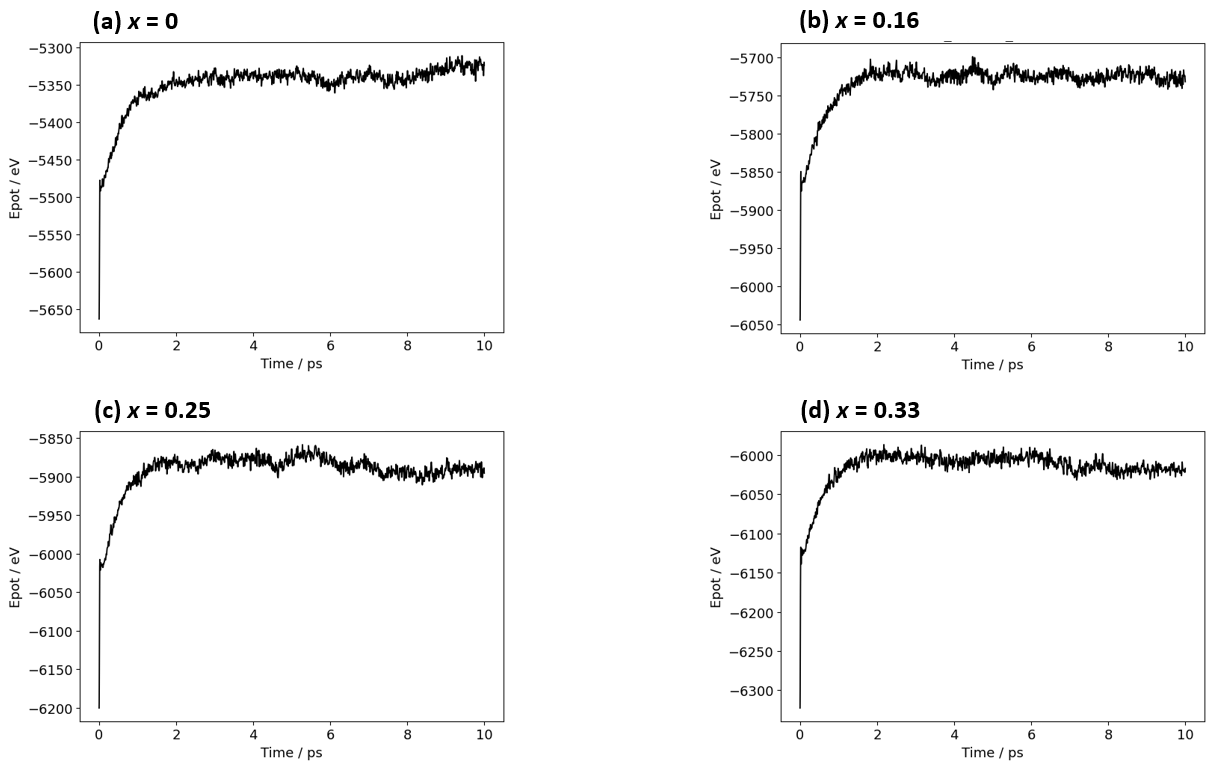


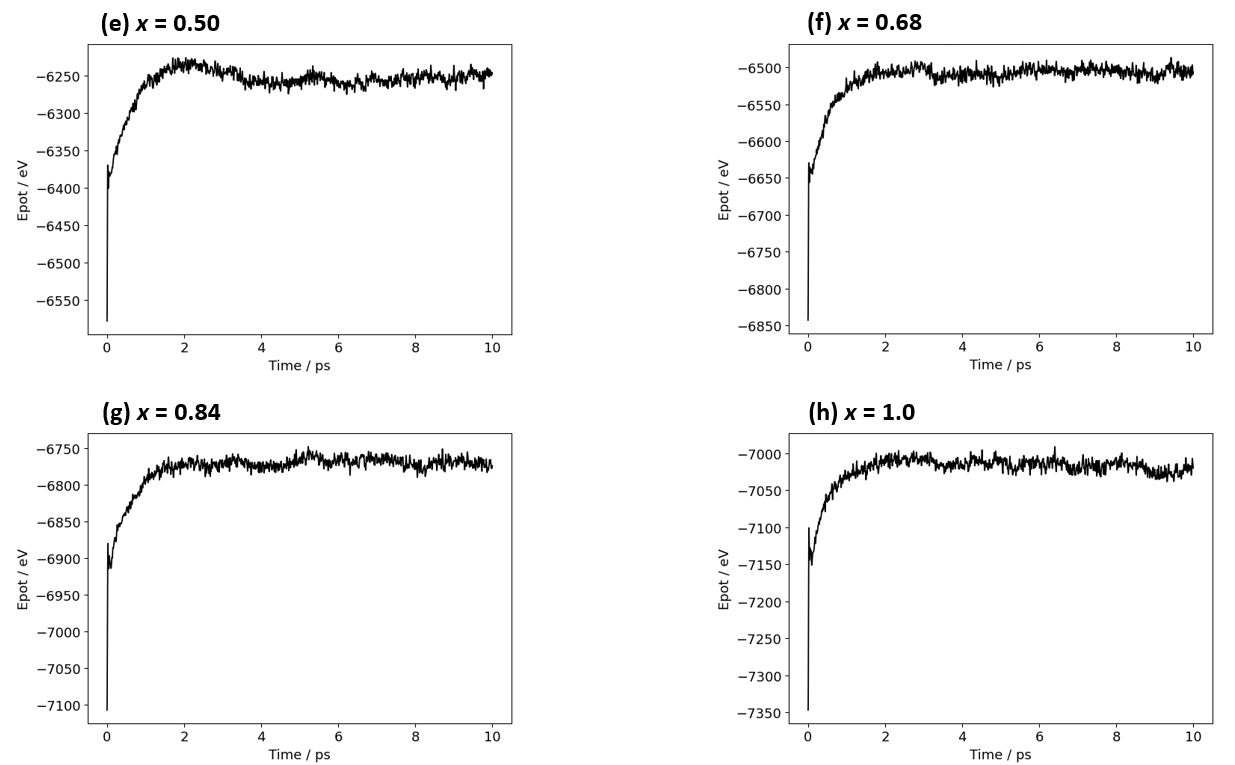


**Figure S3.** Early-time zoom of potential energy profile as a function of MD time (0–10 ps) at 1600 K for various compositions, *x* = 0, 0.16, 0.25, 0.33, 0.50, 0.68, 0.84, and 1.0 shown in Figure S2. These traces support the 200 ps equilibration discard used for MSD fitting.
